# Prophylaxis in Obstetrical Practice1Read before the Alumni Association of the Medical Department of the University of Buffalo, April 25, 1899.

**Published:** 1899-07

**Authors:** William G. Taylor

**Affiliations:** Buffalo, N. Y.


### PROPHYLAXIS IN OBSTETRICAL PRACTICE.[1]

By WILLIAM G. TAYLOR, M. D., Buffalo, N. Y.

THERE is no subject in the whole domain of medicine or surgery that so claims our best effort as obstetrics, and we should all appreciate our responsibility when we accept a case for attendance. It is a sacred duty that we are called upon to perform, a duty which too many of us enter upon carelessly, without thinking what it means to shoulder such responsibilities.

When a young woman comes to any one of us for care during confinement, she comes with all the confidence and assurance possible. She comes oftentimes with rosy cheeks and full of life, in other words, the picture of health. She looks into the future with a cheerful heart in anticipation of being a mother and enjoying the blessings that come with motherhood. But, alas! how often we see later the same woman more or less a physical wreck. Her color and vivaciousness have given way to pallor and lassitude. Her face shows signs of pain and suffering, perhaps from salpingitis or the discomfort of a lacerated perineum. She has lost, in a sense, the pride she once had by virtue of her now shattered nervous system.

It is with the above picture in mind that this paper was suggested to me. In the time allotted to mê I can only speak of a few points.

The items that I will dwell upon are familiar to ail of ,you and my idea is, not to tell you something that you don't know, but rather to bring out a discussion of this important subject.

An early engagement to attend a woman in labor is a satisfaction to every physician and women should be encouraged in it and told of the dangers that may arise by delay. The family history and a physical examination are important in every case, and of a woman who has borne children a careful inquiry should be made concerning her previous pregnancies and former labors. Patients often ask, "Doctor, what shall I do to prepare for my confinement?" I say to them, "Live naturally. Live as you always have lived, but give special attention to food. See that it is plain and wholesome. Drink freely of water. As to clothing, hang skirts freely from shoulders and wear underwraps summer and winter. Avoid any pressure on nipples and abdomen from corsets, and the like. As to hygiene, take regular outdoor exercise. Move the bowels daily, or at least, every other day. Keep the skin active by baths and last, but most important of all, being the urine for examination."

---

1. Read before the Alumni Association of the Medical Department of the University of Buffalo, April 25, 1899.

Women seek advice on almost all conditions in pregnancy, but ignore the kidneys, probably because of no specific symptoms. To me it would be criminal neglect not to examine the urine regularly and systematically. One need never be alarmed that a woman is not doing well during her pregnancy, with possibly one or two exceptions, if the urine is what it should be, even though he does not see her from the time of conception to labor at term. This is a strong statement, but very true.

We have all seen a picture of this kind: a woman becomes sleepless and irritable, suffers from headaches, loss of appetite and constipated bowels. Her urine is scanty, high colored and perhaps at this time contains a little albumin. There is edema of eyelids and extremities. What does it mean? It means imperfect elimination and it means that a milk diet, hot air baths, plenty of fluids, bowels and skin rendered active, is the treatment that should be instituted and without delay.

Many physicians give no attention to the quantity of urine excreted in twenty-four hours, or the total solids it contains, the presence of albumin being the only cause for alarm. In my own experience women have had serious trouble without there being even a trace of albumin found in the urine. The total solids, therefore, are the most important consideration and must be tested from a twenty-four hours' sample and if they are found reduced, measures should be taken to increase them. My patients are requested to bring a sample of urine to my office every month after the fourth month until the seventh; then every two weeks until they are confined. If they fail, I notify them by post, if necessary. They usually report before the second notice is sent. Many cases of eclampsia can be prevented if a physician has his patients under his control.

The care of the breasts and nipples is an important consideration which brings us good or ill repute in the estimation of our families. I don't know of a more exasperating condition than an infection occurring through a cracked nipple leading to suppuration. Giving birth to a child is no comparison, women tell me. A great deal has been said and written about the care of nipples previous to confinement. I have lost all faith in any treatment that has thus far been instituted. Remedies, as brandy, witch hazel, and the like, used to harden, make them crack more easily and ointments make them more friable. I have tried both, but find that by leaving them alone, excepting an antiseptic wash for cleanliness, is all that is necessary, and they do just as well. The important part in their care comes after labor.

In giving my directions to the nurse previous to the appearance of milk, I request her to wash off the nipples with a boracic acid solution before each nursing. Put the baby to the nipple for two or three minutes at a time only, first to teach it how to nurse and second, to give it the colostrum. Again, wash the nipples and put on an ointment of urguentine or resorcin in lanoline until the baby nurses again, four or five hours later. I believe more sore nipples are caused by the baby nursing too long at a time than a first thought would suggest. Many a woman allows her baby to tug away for twenty or thirty minutes and the consequence is that she has a sore nipple before the milk comes Another thing that I want to condemn is the indiscriminate use of breast pumps. There is always a disposition on the part of the mother, and especially the nurse, just as soon as the breasts get hard and somewhat painful, which usually happens on the third day, to use the breast pump.

I believe the use of a breast pump at this time is bad practice, is a frequent cause of sore nipples and of injury and inflammation of the breast. We must remember that the first fulness in the breast is not caused by milk, but by blood. The breast is engorged with blood to the fullest extent and a breast pump only increases that congestion. Rather pad the breast well with cotton, put on a wide bandage, having previously rubbed it with hot castor oil. I speak of castor oil particularly because of its being more astringent than other oils. The idea is not to rub hard to milk out the breast, but just keep the hand moving over the breast lightly, not for half an hour, but for two or three hours, if necessary. Pretty soon the tension gives way to comfort and the next day's milk takes the place of the congestion.

Another point that I will speak of in the line of prophylaxis is the care of a woman during and after labor. It is needless to say that the obstetrician should be cleanly. That question has been ground into us so thoroughly that we consider it old. The man who does not heed its teaching is a failure as an accoucheur. But the question of how much preparation our patients need before being examined by one who has thoroughly cleansed his hands, is one upon which we may not all agree. It has been said that the vagina in a pregnant woman is sterile. If that is true, why do so many advise the bichloride douche before the first examination, or douches during the puerperium? I will not pretend to answer that question, for all I know about it is what I hear and read, but I have had as uniformily good results where I have left the vagina alone as where I

have scrubbed it out with green soap and water, followed by a bichloride solution. I do recommend the bichloride douche, however. The hair should be cut away from the ostium vaginæ and the genitals thoroughly cleansed and the inside of the thighs as well, to prevent carrying infection into the vagina from without.

### THE PREVENTION OF POST PARTUM HEMORRHAGE.

The old saying "an ounce of prevention is worth a pound of cure" has proven itself again and again in the failure to carry out certain details in the third stage of labor. A uterus that has labored faithfully for sixteen hours or more, is lacking in muscular tone and the utmost care should be taken to secure its firm contraction after the birth of the child. I believe that post partum hemorrhage is largely a preventable condition and we should assist nature by patient waiting, if necessary, to secure its contraction.

The method of Crédé in dealing with the placenta is followed more or less generally by most physicians and it is to be commended. After the birth of the child the uterus should be immediately grasped by the nurse or assistant and held until the delivery of the after-birth and even for a half hour afterward would be good practice. There is no ironclad rule that can be laid down as to how soon the placenta should be delivered, but it should not be less than twenty minutes, and even thirty or forty or longer, if the uterus shows signs of relaxation. I usually wait until the patient has had two or three natural pains which show a natural tone upon the part of the uterus. If chloroform has been given for any prolonged period, the above treatment is especially needful.

A hot bichloride douche should be given after the delivery of the placenta and its membranes, first as a hemostatic and to remove clots, and secondly, for its antiseptic effect. The patient's condition should be watched if the pulse is over 100. Indiscriminate douching after labor is not good practice. Unless one has a competent nurse, I believe a daily douche often does more harm than good. If, however, there is an offensive lochia it should be given under strict antiseptic precautions. The vagina should be treated as an open wound and closed with an antiseptic pad.

After the first twelve hours the patient may sit up to urinate, unless there is a tendency to hemorrhage or other complications, as the position favors drainage and does away with any vesical irritation from the use of the catheter. The use of the catheter is, however, necessary in some cases. The patient can at this time move more actively in bed and change her position from side to side to prevent retroversions that are so common after labor. The last point is important.

### REPAIR OF LACERATIONS.

I was taught that to have a laceration was a mark of poor workmanship, but I have noticed as I have grown older in practice that it happens now and again to the most experienced, and I have concluded that it is not such an unpardonable sin. In my early practice I was inclined to overlook slight tears and to think them of no consequence, but for some years I have examined my patients after labor most carefully and find oftentimes tears that only a minute examination could determine. I consider any physician who examines his patient carefully after labor to find tears and repair them should they exist, not a weak physician, but a strong one.

Much can be done to save the perineum. I find that a bichloride towel folded and placed over the perineum with the thumb on the head, using pressure and keeping it well adapted to the outlet saves the perineum a great deal of mechanical pressure from the hand and consequent laceration. Episiotomy has saved me many a tear and I always make it, if I think there is any danger of not delivering the head safely. The parts always heal kindly and can be sewed without the help of an assistant. All tears, however slight, should be repaired, even to a split in the mucous membrane, to prevent infection and to ensure the return of the parts to their healthy condition.

To summarise the points in this paper, which I have attempted to emphasize: (1) Family and personal history; (2) General physical examination; (3) General hygiene as food, clothing, etc.; (4) Special attention to urine; (5) Care of nipples and breasts; (6) Asepsis and douching before and after labor; (7) Prevention of post partum hemorrhage; (8) Repair of lacerations.

### DISCUSSION.

Dr. MATTHEW D. MANN—The paper read by Dr. Taylor is so eminently sensible, practical and up-to-date as to leave very little to be said on the subject. I agree with him in almost every detail, but can perhaps add a few points that he has not touched and emphasize a few that he has. One is with regard to exercise. I believe that the pregnant woman should take as much exercise as she can, but that amount is a question very hard to settle. Some can take a great deal, while others are so incapacitated, during the later months—especially when they have previously borne children and the heavy abdomen falls forward—that they can do hardly anything. In such cases a properly constructed abdominal support will enable them to get about.

With regard to bicycling: I have always advised women who have been accustomed to ride to keep right on and have known them to ride during the first four or five months without any harm whatever, but with manifest benefit. With regard to the kidneys I wish to emphasize what the doctor has said. There may be a considerable amount of albumin in the urine without any evidence of serious trouble. I think that some authorities state that as many as 25 per cent. of pregnant women have albumin in the urine. Certainly there are no serious evidences of eclampsia in that number. What he has said with regard to the total amount of urine is of vastly more importance. A woman may have very serious trouble without any albumin being present, the only symptom being a renal insufficiency, which means that certain poisons are retained which ought to be gotten rid of and that she is suffering from an auto-intoxication, which may result in the gravest manifestations. I believe this to be the cause of a great many of those cases of intractable nausea and vomiting of pregnancy. I feel very sure that it is the cause, judging from the results of treatment. You will find in all these cases that the total amount is very small; they are vomiting and can take very little water, so the amount excreted is in proportion to the amount of fluids ingested. I believe the renal insufficiency antedates the severe nausea; they may have had some, stopped taking water and the renal insufficiency becomes marked, followed by symptoms of auto-intoxication. I have seen a number of cases that have been greatly benefited by the forced injection of fluids by way of the rectum. If we can improve the action of the kidneys they will recover more quickly than by any other means. Moreover, I believe there is a better chance of saving the life of the child under this line of treatment.

What Dr. Taylor has said in regard to the nipples and breasts is exactly in accordance with what I have long believed, practised and taught—to let the breasts largely alone, refrain from too much rubbing, the use of breast pumps, and the like, most of them being of no earthly use. It requires an intermittent suction, as a steady suction is not natural and will not answer the purpose. Putting cotton on them and bandaging them firmly gives better results than working at them. We should always remember that the so-called broken breast is the result of an infection entering via the nipple and milk ducts and that it is a late manifestation. It does not occur during the first week with the rush of milk. The lumps which occur at that time soon disappear, when the child begins to nurse and we can afford to let them alone. If we see that the nipples do not get sore the danger of abscess is very greatly diminished. I do not mean the little abscesses, which start as a pimple on the skin, but mastitis.

With regard to puerperal infection, I think the doctor is right, and that very few cases start from within. Examinations made by many bacteriologists, notably by Dr. W. Williams, of Johns Hopkins, who shows that the vagina is absolutely sterile; that previous observations were faulty; that in an examination of over 100 cases he found the vagina sterile almost every time. Whence then does the infection come? Undoubtedly from the examining fingers. The hands of the accoucheur should be as thoroughly cleansed as for an abdominal section. It is possible that infection may arise from pus tubes, antedating the pregnancy. One tube may be practically normal, the other full of pus. I have seen a case where gonorrhea was acquired during the pregnancy. The mother had severe puerperal septicemia and the child ophthalmia neonatorum from an infection antedating the labor. Danger also arises from the external genitals and they should be carefully cleansed.

As to douches afterward, I think we can dispense with them. In one of the foreign hospitals 1,000 cases were treated thus and the same number without them; the second series did better than the former. We can let them alone as a matter of routine practice, where there is no decided indication.

With regard to sitting up, I remember Dr. Goodell's advice, which was eminently sound. He made all his women sit up on the fifth day. He had them get up to empty the bladder after the first twenty-four hours; and said there was a form of puerperal sepsis which was cured by getting up and allowing clots to escape from the vagina

Lacerations, I believe, as Dr. Taylor has stated, should be attended to at once. I believe that there is sometimes such a disproportion between the head and the vagina that a laceration is bound to occur. Whether severe or slight, I believe they should be sewed up.

